# Self-reported prevalence of atherothrombosis in a general population sample of adults in Greece; A telephone survey

**DOI:** 10.1186/1471-2261-11-16

**Published:** 2011-04-14

**Authors:** Nikos Maniadakis, Georgia Kourlaba, Vasileios Fragoulakis

**Affiliations:** 1 Department of Health Services Management, National School of Public Health, Athens, Greece; 2 Department of Epidemiology and Biostatistics, National School of Public Health, Athens, Greece

**Keywords:** atherosclerosis, atherothrombotic risk factors, coronary artery disease, cerebrovascular disease

## Abstract

**Background:**

The aim of this study was to estimate the prevalence of selected atherothrombotic risk factors and several clinical manifestations of atherothrombosis, as well as the utilization rates of selected vascular interventions in Greece.

**Methods:**

During December 2009, 3,007 adults (aged 47 ± 16 years, 48.3% men and 51.7% women) recruited in a random-digit dialed telephone survey (response rate: 16%). The sample size was selected following a multistage and stratified by gender, age group, and Greek region procedure in order to be more representative. Data regarding medical history and socio-demographic characteristics of the participants were collected.

**Results:**

Overall, 6.5%, 17.7% and 14.0% of participants reported that they had been diagnosed with diabetes mellitus, hypertension and hypercholesterolemia, respectively. In the overall sample, 2.5% of participants reported that they had been diagnosed with angina, 2.0% with myocardial infarction, 1.6% with stroke and 2.5% with peripheral artery disease. Overall, 1.5% of participants reported that they had undergone percutaneous coronary intervention, 1.4% coronary artery bypass grafting, 0.6% angioplasty of a peripheral vessel, and 0.7% surgery of a peripheral vessel.

**Conclusion:**

Despite the limitations may occur due to the sampling procedure, the findings of the present study indicate that atherothrombosis affects a large portion of the population in Greece and it is expected to impose a significant economic burden. The data of the current study could contribute in obtaining an accurate estimation of the economic burden of atherothrombosis in Greece because people who are aware of their condition/disease are those who use health care resources.

## Background

Atherothrombosis is defined as an unpredictable, sudden disruption of an atherosclerotic plaque, which leads to platelet activation and thrombus formation. Atherothrombosis is the underlying condition which causes coronary artery disease (CAD), including myocardial infarction (MI) and angina pectoris (stable or unstable), stroke, and peripheral artery disease (PAD)[1]. CAD and stroke constitute the leading causes of death and disability worldwide [2]. Moreover, a recently published systematic cost-of-illness study that aimed to assess the economic burden of cardiovascular disease (CVD) in the enlarged European Union showed that CVD is a leading public health problem [3]. However, the major limitation of the aforementioned study is the unavailability of necessary data which caused the authors to make many assumptions and extrapolations.

In Greece, an accurate estimation of the total economic cost of atherothrombosis has never been provided due to lack of data. This highlights the need to collect data in order to obtain an accurate estimation of the total economic burden of atherothrombotic disease. The data required for this purpose is the prevalence of several clinical manifestations of the disease and several atherothrombotic risk factors as well as the utilization rate and cost of various health services related to the atherothrombosis management.

Age, smoking, hypertension, diabetes mellitus and hypercholesterolemia are major atherosclerotic risk factors. As concerns the prevalence of the last three risk factors, few data are available in Greece [4-11]. However, the proportion of people who are aware of these conditions and not the actual prevalence, which often includes silent cases of people who are not consuming resources, is necessary in order to achieve an accurate estimation of the economic burden of atherothrombotic disease in a country.

In Greece, there are only few data regarding the awareness and treatment of the aforementioned risk factors. Moreover, there are limited data regarding the prevalence of the clinical manifestations of atherothrombosis [12-15], while data regarding the utilization rates of several vascular interventions (i.e. percutaneous coronary intervention (PCI), coronary artery bypass grafting (CABG), angioplasty or surgery of a peripheral vessel) are lacking. Therefore, the objective of the current study was to evaluate the prevalence of selected atherothrombotic risk factors and clinical manifestations of atherothrombosis, as well as the utilization rates of vascular interventions in a representative nationwide sample of Greek adults. These data will be used in order to estimate the total economic burden of atherothrombotic disease in Greece.

## Methods

### Study sample

During December 2009, 89,526 phone numbers were randomly selected in order to recruit men and women from all Greek regions to participate in a random-digit dialled (RDD) telephone nationwide survey. From the study design, it had been decided that the sample would be stratified by gender, age group and Greek region (i.e. proportionally stratified sampling procedure) in order to be more representative. Moreover, it had been decided that 0.03% of the total population of each Greek region, with only one person per household, would be interviewed in order to obtain 3,000 completed questionnaires (Additional file 1). This sample size had been calculated as adequate in order to estimate the proportions with a maximum error of 2% at a probability level < 0.05. The procedure used to recruit the sample was the computer assisted telephone interviewing (C.A.T.I.) technique. In particular, telephone numbers were loaded in a phone centre and were distributed to agents on availability basis. When a call resulted to an answer the questionnaire appeared to the agent and the communication was initiated. All agent and campaign activities were monitored by supervisors through performance - monitoring software. From the initial planning, several routines were implemented during the dialling process in order to minimize the non-response rate. From 89,526 phone numbers, only 41,639 were handled calls (conducted persons). The rest being rejected due to various reasons (No answer, busy, etc). Of the 41,639 conducted persons, 18,758 were eligible for the present study. Persons were unsuitable for interviewing only if they were younger than 18 years old, or they belonged to a combination of age group-gender-region (i.e. strata) for which the necessary number of participants had been enrolled. Of 18,758 eligible subjects, only 3,007 agreed to participate (response rate: 16.0%). The average time of completion of a questionnaire was 3 minutes and 21 seconds. All participants were informed by the interviewer about the aims and procedures of the study. However, since this was a telephone interview no written informed consent was obtained from the participants. The study was approved by the ethical committee of our Institution and was carried out in accordance with the Declaration of Helsinki (1989) of the World Medical Association.

### Investigated measurements

A structured questionnaire (Additional file 1) that was developed specifically for the purposes of the current study was used in order to retrieve various data from participants. In particular, participants were asked to report their age (divided into 5 groups: 18-24, 25-39, 40-54, 55-64, 65+ years old), marital status (married/cohabiting, single, divorced/separated, and widowed), educational status (years of education), nationality (Greek, and immigrants), region of residence (urban, rural), and occupational status (unemployed, retired, university student, housewife, self employed, servant, farmer, and unable to work). In addition, participants were asked to report their weight (kg) and height (m) so that their body mass index (BMI) could be determined. Then, participants were categorised as "normal-weight" (18.5 < BMI < 25 kg/m^2^), "overweight" (25 < BMI < 29.9 kg/m^2^) and "obese" (BMI > 29.9 kg/m^2^). Participants' smoking habits were also recorded (i.e. never smokers, former smokers and current smokers).

Information relevant to health status, with a focus on arterial hypertension (defined as diagnosed by doctor or use of anti-hypertensive treatment), hypercholesterolemia (defined as diagnosed by doctor or use of lipid lowering agents) and diabetes mellitus (defined as diagnosed by doctor or use of hypoglycemic treatment), as well as the time of the first diagnosis, were recorded. Any history of angina, MI, stroke, or PAD diagnosed by a doctor, as well as the time of first diagnosis were also recorded. Finally, participants were asked to report whether and when they had undergone any of the following interventions: PCI, CABG, angioplasty of a peripheral vessel or surgery of a peripheral vessel.

### Statistical analysis

Categorical variables are presented as absolute (n) and relative frequencies (%). The chi-square test without the correction of continuity was used in order to evaluate the associations between categorical variables. The prevalence of one, two, and three or more atherothrombotic risk factors was determined for the overall study population and men and women, separately. Then, the multiple multinomial logistic regression analysis was conducted in order to evaluate the association of several socio-demographic factors (independent variables) with the probability of having 0, 1 or ≥2 atherothrombotic risk factors (dependent variables). The results are presented as odds ratios (OR) and 95% confidence interval (CI).

A probability value of 5% was considered as statistically significant. All statistical calculations were performed using SPSS version 17.0 software (SPSS Inc, Chicago, Il, USA).

## Results

The socio-demographic characteristics of the participants are presented in Table 1. It was observed that the majority of participants were married, Greek, retired and servants, overweight/obese, non-smokers and resided in urban areas. Moreover, it should be highlighted that almost 1/5 of participants reported that they had a very low educational status (≤6 years) and almost 6% of participants reported that they were unemployed.

**Table 1:** Population characteristics (N = 3,007)

Characteristics	n (%)
Gender	
*Male*	1,451 (48.3%)
*Female*	1,556 (51.7%)
Age (in years)	
*18-24*	277 (9.2%)
*25-39*	905 (30.1%)
*40-54*	807 (26.8%)
*55-64*	378 (12.6%)
*65+*	640 (21.3%)
Marital status	
*Married/living together*	2,089 (69.5%)
*Single*	672 (22.4%)
*Divorced/separated*	71 (2.4%)
*Widowed*	172 (5.7%)
Area of residence	
*Urban*	2,185 (72.7%)
*Rural*	822 (27.3%)
Nationality	
*Greek*	2,919 (97.1%)
*Other*	88 (2.9%)
Occupational status	
*Unemployed*	171 (5.7%)
*Retired*	694 (23.1%)
*University students*	152 (5.1%)
*Housewife*	436 (14.5%)
*Servant*	859 (28.6%)
*Self-employed*	591 (19.7%)
*Farmer*	97 (3.2%)
BMI	
*Normal weight*	1,270 (43.0%)
*Overweight*	1,201 (40.7%)
*Obese*	483 (16.3%)
Educational status	
*Very low (*≤*6*)	612 (20.4%)
*Low (6-12)*	1,183 (39.5%)
*Moderate (12-14)*	155 (5.2%)
*High (14-16)*	941 (31.4%)
*Very high (>16)*	108 (3.6%)
Smoking habits	
*Non-smokers*	1,919 (63.8%)
*1-20 cigarettes*	750 (24.9%)
*21+*	338 (11.2%)

BMI: Body Mass Index

The prevalence of the clinical manifestations of atherothrombosis in relation to participants' socio-demographic characteristics is presented in Table 2. In the overall sample, 2.5% of participants reported that they had been diagnosed with angina, 2.0% with MI, 1.6% with stroke and 2.5% with PAD. The prevalence of all manifestations increased with increasing age. The prevalence of angina, stroke, and PAD was significantly higher among widowed people, while the prevalence of MI was significantly higher in widowed, divorced/separated and married compared to singles. Furthermore, the prevalence of these diseases was higher among retired people, obese, those with very low educational status, and non-smokers.

**Table 2:** The prevalence of clinical manifestations of atherothrombosis (N = 3,007)

	Angina	Myocardial infarction	Stroke	Peripheral arterial disease
Total population	2.5%	2.0%	1.6%	2.5%
Gender				
*Male*	6.3%*	3.2%*	1.8%	2.2%
*Female*	2.8%	1.0%	1.4%	2.7%
Age (in years)				
*18-24*	0.0%*	0.0%*	0.0%*	0.0%*
*25-39*	0.6%	0.1%	0.3%	0.2%
*40-54*	3.7%	1.2%	0.4%	1.6%
*55-64*	4.8%	2.1%	2.4%	3.7%
*65+*	13.0%	6.7%	5.2%	7.0%
Marital status				
*Married/living together*	5.4%*	2.6%*	1.6%*	2.5%*
*Single*	0.9%	0.3%	0.6%	0.2%
*Divorced/separated*	5.6%	2.8%	0.0%	2.8%
*Widowed*	8.1%	2.3%	5.8%	10.5%
Area of residence				
*Urban*	4.4%	2.1%	1.7%	2.3%
*Rural*	5.0%	2.0%	1.3%	2.9%
Nationality				
*Greek*	4.6%	2.1%	1.6%	2.5%
*Other*	1.1%	1.1%	0.0%	2.3%
Occupational status				
*Unemployed*	1.8%*	0.0%*	0.0%*	0.6%*
*Retired*	12.4%	6.6%	5.6%	6.3%
*University students*	0.0%	0.0%	0.0%	0.0%
*Housewife*	3.0%	0.7%	1.2%	2.3%
*Servant*	1.9%	0.7%	0.2%	0.7%
*Self-employed*	2.2%	0.9%	0.0%	1.9%
*Farmer*	4.1%	1.0%	1.0%	2.1%
BMI				
*Normal weight*	2.3%*	0.8%*	0.9%*	1.1%*
*Overweight*	4.7%	2.2%	1.9%	2.6%
*Obese*	9.1%	4.8%	2.7%	5.6%
Educational status				
*Very low (*≤*6*)	10.0%*	4.6%*	3.6%*	6.4%*
*Low (6-12)*	3.1%	1.4%	1.4%	1.6%
*Moderate (12-14)*	2.6%	0.7%	0.0%	0.7%
*High (14-16)*	3.5%	1.6%	1.0%	1.6%
*Very high (>16)*	0.9%	0.9%	0.0%	0.0%
Smoking habits				
*Non-smokers*	5.6%*	2.7%*	2.1%*	2.9%
*1-20 cigarettes*	2.5%	1.2%	0.7%	1.5%
*21+*	3.0%	0.6%	0.9%	2.1%

* p-value < 0.05

BMI: Body Mass Index

The prevalence of several atherothrombosis-related interventions in relation to participants' socio-demographic characteristics is presented in Table 3. Overall, 1.5% of participants reported that they had undergone PCI, 1.4% CABG, 0.6% angioplasty of a peripheral vessel and 0.7% surgery of a peripheral vessel. The associations observed between the prevalence of these interventions and participants' characteristics were similar to those observed between atherothrombotic clinical manifestations and participants' characteristics.

**Table 3:** The prevalence of several atherothrombosis-related interventions (N = 3,007)

	PCI	CABG	Angioplasty of peripheral vessel	Surgery of peripheral vessel
Total population	1.5%	1.4%	0.6%	0.7%
Gender				
*Male*	2.6%*	2.5%*	0.6%	0.6%
*Female*	0.5%	0.4%	0.6%	0.8%
Age (in years)				
*18-24*	0.0%*	0.0%*	0.0%	0.0%*
*25-39*	0.2%	0.0%	0.2%	0.0%
*40-54*	0.9%	0.9%	1.0%	0.4%
*55-64*	2.7%	1.1%	0.8%	1.1%
*65+*	4.1%	4.8%	0.9%	2.2%
Marital status				
*Married/living together*	2.0%*	1.7%	0.7%	0.7%*
*Single*	0.3%	0.3%	0.2%	0.0%
*Divorced/separated*	1.4%	1.4%	0.0%	0.0%
*Widowed*	0.6%	1.7%	1.7%	3.5%
Area of residence				
*Urban*	1.4%	1.4%	0.6%	0.5%*
*Rural*	1.7%	1.3%	0.6%	1.2%
Nationality				
*Greek*	1.5%	1.4%	0.6%*	0.7%
*Other*	0.0%	1.1%	2.3%	0.0%
Occupational status				
*Unemployed*	0.6%*	0.0%*	0.6%	0.0%*
*Retired*	4.3%	4.9%	1.2%	1.7%
*University students*	0.0%	0.0%	0.0%	0.0%
*Housewife*	0.5%	0.5%	0.5%	0.9%
*Servant*	0.7%	0.2%	0.2%	0.2%
*Self-employed*	0.5%	0.5%	1.0%	0.3%
*Farmer*	2.1%	1.0%	0.0%	1.0%
BMI				
*Normal weight*	0.7%*	0.9%*	0.4%*	0.4%*
*Overweight*	1.7%	1.1%	0.5%	0.6%
*Obese*	2.9%	3.5%	1.7%	1.5%
Educational status				
*Very low (*≤*6*)	3.4%*	2.6%*	1.3%	1.5%
*Low (6-12)*	0.9%	0.9%	0.5%	0.7%
*Moderate (12-14)*	0.7%	0.7%	0.0%	0.0%
*High (14-16)*	1.2%	1.5%	0.5%	0.4%
*Very high (>16)*	0.9%	0.0%	0.0%	0.0%
Smoking habits				
*Non-smokers*	1.9%	1.8%*	0.6%	0.9%
*1-20 cigarettes*	0.9%	0.7%	0.8%	0.3%
*21+*	0.6%	0.6%	0.6%	0.6%

* p-value < 0.05

PCI: Percutaneous Coronary Intervention,

CABG: Coronary Artery Bypass Grafting

BMI: Body Mass Index

The distribution of selected traditional CVD risk factors in relation to participants' socio-demographic characteristics is presented in Table 4. Overall, 6.5%, 17.7% and 14.0% of participants reported that they had been diagnosed with diabetes mellitus, hypertension and hypercholesterolemia, and/or being treated for these conditions. The prevalence of these atherothrombotic risk factors was significantly higher among participants aged more than 65 years old, widowed, Greek, retired, obese, those with very low educational status and non-smokers. Moreover, although no difference was detected in the prevalence of diabetes mellitus between men and women, women were more likely to be hypertensive and hypercholesterolemic.

**Table 4:** The prevalence of selected cardiovascular disease risk factors (N = 3,007)

	Diabetes mellitus	Hypertension	Hypercholesterolemia
Total population	6.4%	17.7%	14.0%
Gender			
*Male*	6.3%	15.7%*	11.4%*
*Female*	6.5%	19.5%	16.4%
Age (in years)			
*18-24*	0.7%*	0.0%*	0.7%*
*25-39*	0.8%	2.0%	2.5%
*40-54*	4.3%	11.9%	13.0%
*55-64*	10.9%	32.5%	25.9%
*65+*	16.7%	45.9%	30.2%
Marital status			
*Married/living together*	7.1%*	20.0%*	16.4%
*Single*	1.5%	3.0%	2.8%
*Divorced/separated*	4.2%	15.5%	16.9%
*Widowed*	18.0%	47.7%	27.9%
Area of residence			
*Urban*	6.8%	16.4%	13.4%
*Rural*	5.4%	21.1%	14.8%
Nationality			
*Greek*	6.4%	18.1%*	14.4%*
*Other*	5.7%	3.4%	2.3%
Occupational status			
*Unemployed*	3.5%*	4.7%*	6.4%*
*Retired*	16.1%	43.1%	29.7%
*University students*	0.7%	0.7%	1.3%
*Housewife*	6.0%	22.7%	18.4%
*Servant*	2.2%	6.4%	7.2%
*Self-employed*	3.6%	8.3%	7.6%
*Farmer*	6.2%	19.6%	15.5%
BMI			
*Normal weight*	3.2%*	7.8%*	7.6%*
*Overweight*	6.5%	20.7%	17.0%
*Obese*	14.3%	34.8%	22.8%
Educational status			
*Very low (*≤*6*)	15.9%*	41.2%*	27.3%*
*Low (6-12)*	4.0%	13.2%	11.5%
*Moderate (12-14)*	5.2%	3.2%	7.7%
*High (14-16)*	4.0%	11.4%	10.5%
*Very high (>16)*	1.9%	8.3%	6.5%
Smoking habits			
*Non-smokers*	7.7%*	21.6%*	16.1%*
*1-20 cigarettes*	4.1%	10.4%	10.5%
*21+*	4.1%	11.3%	9.8%

*p-value < 0.05

BMI: Body Mass Index

Considering clusters of smoking, diabetes mellitus, hypertension and hypercholesterolemia, it was found that 48% of participants had none of these risk factors, 40% had one, 9.5% had two and 2.5% had three or more risk factors. Including obesity in the aforementioned risk factors, 41.4% of participants were found to have none, 40.7% to have one, 13.4% to have two and 4.7% to have three or more risk factors. A statistically significantly difference was detected in the distribution of number of atherothrombotic risk factors between genders. In particular, women were more likely to have three risk factors or more (6%) compared to men (3.5%, p = 0.002).

Then, multinomial logistic regression analysis was carried out to evaluate the association of selected socio-demographic risk factors with the probability of having one or two and more risk factors among the five risk factors (obesity, smoking, diabetes mellitus, hypertension, hypercholesterolemia). This analysis revealed that participants' age, educational status and gender were significantly associated with the probability of having one risk factor versus none, while only age and educational status were associated with the probability of having two or more risk factors versus none (Table 5).

**Table 5:** Factors associated with having one or two and/or more atherothrombotic risk factors*

	One risk factor vs. none		Two or more risk factors vs. none	
**Characteristics**	**OR**	**95% CI**	**OR**	**95% CI**

Gender				
*Male*	Reference	-	Reference	-
*Female*	**0.80**	**0.68 - 0.94**	0.89	0.72 - 1.10
Age (in years)				
*18-24*	Reference	-	Reference	-
*25-39*	1.17	0.89 - 1.55	**2.81**	**1.25 - 6.28**
*40-54*	**1.54**	**1.15 - 2.05**	**11.09**	**5.06 - 24.27**
*55-64*	**2.18**	**1.53 - 3.10**	**20.25**	**9.00 - 45.54**
*65+*	**1.31**	**0.94 - 1.83**	**14.48**	**6.51 - 32.20**
Area of residence				
*Urban*	Reference	-	Reference	-
*Rural*	0.84	0.70 - 1.01	0.80	0.63 - 1.02
Educational status				
*Very low (*≤*6*)	Reference	-	Reference	-
*Low (6-12)*	1.04	0.80 - 1.36	**0.65**	**0.48 - 0.88**
*Moderate (12-14)*	1.08	0.70 - 1.65	0.70	0.38 - 1.30
*High (14-16)*	0.89	0.67 - 1.16	**0.45**	**0.32 - 0.63**
*Very high (>16)*	**0.64**	**0.40 - 1.00**	**0.27**	**0.12 - 0.60**

* Hypertension, hypercholesterolemia, diabetes mellitus, smoking and obesity were considered

CI: Confidence Interval; BMI: Body Mass Index

## Discussion

The present study provided data on the self-reported prevalence of CVD risk factors, main clinical manifestations of atherothrombosis and utilization of vascular interventions at a nationwide sample of 3,007 adults from Greece, in 2009. Although a considerable number of previous studies have presented similar data, they have been conducted earlier than 2005 [4-8,10,16-18]. Therefore, there are no recent data regarding the prevalence of atherothombosis in Greece. Moreover, the preset study was conducted as a part of a cost-of-illness study (The Burden of Atherothrombosis in Greece: Study of Medical and Social Implications) in which almost 800 patients with a history of or at risk for atherothrombosis were followed-up for a period of one year. The aim of these studies was to combine the prevalence data of the telephone survey with the annual cost per patient obtained from the cost-of-illness study in order to estimate the total burden of atherothrombosis in Greece. The findings of the current study indicate that the prevalence of hypertension, hypercholesterolemia, and diabetes mellitus were 17.7%, 14.0% and 6.4%, respectively. Moreover, the prevalence of CAD, stroke and PAD was found to be 4.5%, 1.6% and 2.5%, respectively.

Although the results of the present study are not comparable with those reported in the majority of the previous studies, their findings as well as their sample size, age of population, period of study, etc are presented in Table 6, for the purpose of discussion. The majority of these studies are large-scale epidemiological and well-organized surveys. The comparison shows that the prevalence of any condition/disease in our study was lower than in the others studies [4-11,14-18], with the exception of the Nutritional and Health Survey that was conducted with a similar study design to the one used in the present study [4]. In particular, the prevalence of hypertension, diabetes mellitus and hypercholesterolemia reported in the Nutritional and Health Survey carried out by Pitsavos et al. in 2004, was 15.5%, 6.0% and 19.1%, respectively [4].

**Table 6:** Study design and findings of studies evaluating the prevalence of traditional cardiovascular disease risk factors and clinical manifestations of atherothrombosis in Greece

Risk factor or clinical manifestation of atherothrombosis	Data collection period	Study	Age range of participants	Study sample size	Study design	Method used for identifying disease/condition	Prevalence	Awareness	Treated
**Hypertension**									

	2001-2002	ATTICA [11]	>18 years	2,282	Observational study in province of Attica (random-multistage sampling method)	Arterial blood pressure was measured	31%	13.4%	
	2004	A Nutrition and Health Survey in Greece [4]	18-74 years	5,003	Nationwide random-digit dialled telephone survey	Self-reported hypertension	15.5%		
	1995-2000	EPIC [8]	20-86 years	26,913	Volunteers	Arterial blood pressure was measured	44.7%	24.3%	20.0%
	2002-2004	HYPERTENSHELL [5]	>17 years	11,540	Cross-sectional survey with 98 Health Centers across Greece	Arterial blood pressure was measured	31.1%	18.7%	15.9%
	2004	Naoussa study [7]	15-74 years	1,937	Workers, technicians and clerks of factories of the city of Naoussa	Arterial blood pressure was measured	30.5%	18.6%	11.8%
	1999	Didima study [18]	>18 years	694	Cross-sectional survey in the rural population of Didima village	Arterial blood pressure was measured	28.4%	17.0%	15.5%
	2003-2006	NHANES	>20 years				33.3%	25.8%	22.6%

**Diabetes Mellitus**									
	2001-2002	ATTICA [16]	>18 years	2,282	Observational study in province of Attica (random-multistage sampling method)	Measured glucose levels	6.9%		
	2004	A Nutrition and Health Survey in Greece [4]	18-74 years	5,003	Nationwide random-digit dialled telephone survey	Self-reported diabetes mellitus	6%		
	2002	MEDICAL EXPRESS 2002 [10]	20-94	2,805	Cross-sectional population based survey	Self-reported diabetes mellitus	8.7%		
	2003-2004	The MetS-GREECE Multicenter Study [6]	>18	9,669	Cross-sectional study of a representative sample of Greek adults	Measured fasting glucose levels	10.0%		

**Hypercholesterolemia**									
	2001-2002	ATTICA [17]	>18 years	2,282	Observational study in province of Attica (random-multistage sampling method)	Measured cholesterol levels	37.7%		17%
	2004	A Nutrition and Health Survey in Greece [4]	18-74 years	5,003	Nationwide random-digit dialled telephone survey	Self-reported	19.1%		
	2003-2004	The MetS-GREECE Multicenter Study [23]	>18	4,153	Cross-sectional study of a representative sample of Greek adults	Measured cholesterol and triglycerides levels	62%		

									
**Myocardial infarction**	2002 2006	Gikas et al. [14]	≥20 years	2,805 3,478	Cross-sectional study in Salamis	Self-reported	4.1% 4.8%		
**Coronary Artery Disease**	2003-2004	The MetS-GREECE Multicenter Study [15]	>18	4,153	Cross-sectional study of a representative sample of Greek adults	Physical examination and personal medical history.	9.3%		
**Stroke**	2003-2004	The MetS-GREECE Multicenter Study [15]	>18	4,153	Cross-sectional study of a representative sample of Greek adults	Physical examination and personal medical history.	4.1%		
**Peripheral Artery Disease**	2003-2004	The MetS-GREECE Multicenter Study [15]	>18	4,153	Cross-sectional study of a representative sample of Greek adults	Physical examination and personal medical history.	2.3%		

The differences observed between the findings of our study and those of the previous large-scale epidemiological studies could be attributed to the fact that in the latter, clinical examination has been used to identify subjects suffering from several conditions or diseases. Moreover, comparison of the present results with those of epidemiological studies indicates that the present study seems to underestimate the actual prevalence of the aforementioned risk factors in Greece. However, the current data provide an accurate estimation of the prevalence of people who are aware or/and treated of their condition, since data of epidemiological studies indicate that less than half of sufferers tend to be aware of their condition. For instance, although the prevalence of hypercholesterolemia was estimated to be 37.7% in the ATTICA study, less than 50% of them were aware of their conditions corresponding to about 17% of the total sample [17].

As concerns the prevalence of the several clinical manifestations of atherothrombosis, previous data for Greece are limited. In particular, only one study was found to provide data regarding the prevalence of MI [14]. The results of this study indicate that the prevalence of MI in Greece in 2002 and 2006 was almost twofold higher than was observed in the present study (Table 6). Moreover, the METS GREEK Multicenter study provides some data regarding the prevalence of coronary artery disease, stroke and PAD [15]. With the exception of PAD prevalence which the current results are in accordance with those reported by Athyros et al. the present data seems to underestimate the prevalence of coronary disease and stroke in Greece.

Finally, as concerns the prevalence of the use of several atherothrombosis-related interventions, only limited data are available for Greece. For example, it is known that almost 19,000 PCIs/year are performed, that is 0.2% of the total adult population in Greece undergo a PCI each year [19]. However, our own data indicate that 0.1% of the population underwent PCI during the last year.

Despite the relatively large size of the sample, the results of the present study may be affected by several methodological factors related to the study design. Above all, in conducting a survey by telephone instead of face-to-face at the homes of respondents leads to automatically exclusion of individuals without telephone service [20,21]. Moreover, several studies have showed that the response rate of telephone surveys is lower than that of face-to-face surveys indicating that the former are vulnerable to a greater non-response bias that may affect the representativeness of the sample [20,22]. The response rate of telephone surveys reduces due to low conduct rate that can be attributed to the mobility of people (i.e. individuals are not often at home). It has been showed that around half the initial calls in a typical telephone survey go unanswered. Callbacks - especially those spread across different times and days of the week - are likely to produce a higher response. In the present study these routines were implemented during the dialling process in order to minimize this bias. Moreover, based on initial planning, individuals were unsuitable for interviewing if they belonged to a combination of age group-gender-region (i.e. strata) for which the necessary number of participants had been enrolled, in order to ensure the age-gender-region representativeness of sample. Another factor that leads to lower response rate in a telephone survey is the unwillingness of subjects to cooperate and engage in the interview, as well as the fact that telephone responders are more likely to express dissatisfaction with the length of the interview than face-to-face respondents, despite the fact that the telephone interviews were completed more quickly than the face-to-face interviews [21].

Apart from the low response rate, telephone surveys may suffer from poor response quality compared to face-to-face surveys [21]. First of all, the responder must retrieve all relevant to question information from memory, integrate that informatio [2]n into a summary judgment, and report that judgment accurately. However, a responder may become fatigue, especially when the interview is long and the questions are asked quickly. Moreover, multitasking (i.e. doing several things during interview such as cooking etc) is a phenomenon that may characterize telephone interviews to a greater extent than face-to-face interviews and may affect the quality of responses. Finally, the telephone does not permit responders and interviewers to develop an interpersonal trust as emerges in face-to-face interactions. Consequently, responders may not feel as confident that their interviewers will protect their confidentiality as responders in face-to-face interviews. Moreover, telephone respondents may be less sure of who will have access to their answers and how they might be used, leading these people to be less honest in discussing potentially embarrassing attitudes or behaviors.

## Conclusions

In the present study, the prevalence of atherothrombotic disease, as well as the prevalence of selected traditional atherothrombotic risk factors, was estimated in Greece. These data indicate that atherothrombosis is a prevalent disease in Greece. Therefore, specific programmes aiming to primary prevention of atherothrombosis should be developed and implemented in Greece. Moreover, the data of the current study could contribute in obtaining an accurate estimation of the economic burden of atherothrombosis in Greece because people who are aware of their condition/disease are those who use health care resources. Therefore, this portion is useful to estimate the total direct health care cost related to atherothrombosis at a national level.

## Competing interests

The authors declare that they have no competing interests.

## Authors' contributions

All authors contributed in writing of the manuscript and interpretation of the results, reviewed its content and approved the final version submitted for publication. Furthermore, NM designed the study and supervised data collection; GK contributed to data management and carried out the statistical analysis; VF contributed to data management and preparation of database.

## Funding

The study was sponsored by a grant from Sanofi-Aventis. The study sponsor had no role in the design and conduct of the study; the collection, management, analysis and interpretation of the data; the preparation, approval and submission of the manuscript.

## Pre-publication history

The pre-publication history for this paper can be accessed here:

http://www.biomedcentral.com/1471-2261/11/16/prepub

## Supplementary Material

Additional file 1 **Questionnaire**. The file contains the questionnaire used to collect data. Click here for file

## References

[B1] Viles-Gonzalez JF Fuster V Badimon JJ Atherothrombosis: a widespread disease with unpredictable and life-threatening consequences Eur Heart J 2004 25 1197 207 10.1016/j.ehj.2004.03.011 15246637

[B2] World Health Organization The Atlas of heart disease and stroke Deaths from stroke 2010 http://www.who.int/cardiovascular_diseases/en/cvd_atlas_16_death_from_stroke.pdf

[B3] Leal J Luengo-Fernandez R Gray A Petersen S Rayner M Economic burden of cardiovascular diseases in the enlarged European Union Eur Heart J 2006 27 1610 9 10.1093/eurheartj/ehi733 16495286

[B4] Pitsavos C Milias GA Panagiotakos DB Xenaki D Panagopoulos G Stefanadis C Prevalence of self-reported hypertension and its relation to dietary habits, in adults; a nutrition & health survey in Greece BMC Public Health 2006 6 206 10.1186/1471-2458-6-206 16904009 PMC1559700

[B5] Efstratopoulos AD Voyaki SM Baltas AA Vratsistas FA Kirlas DE Kontoyannis JT Sakellariou JG Triantaphyllou GB Alokrios GA Lianas DN Vasilakis EA Fotiadis KN Kastritsea EE Prevalence, awareness, treatment and control of hypertension in Hellas, Greece: the Hypertension Study in General Practice in Hellas (HYPERTENSHELL) national study Am J Hypertens 2006 19 53 60 10.1016/j.amjhyper.2005.07.011 16461191

[B6] Athyros VG Ganotakis ES Bathianaki M Monedas I Goudevenos IA Papageorgiou AA Papathanasiou A Kakafika AI Mikhailidis DP Elisaf M Awareness, treatment and control of the metabolic syndrome and its components: a multicentre Greek study Hellenic J Cardiol 2005 46 380 6 16422124

[B7] Sarafidis PA Lasaridis A Gousopoulos S Zebekakis P Nikolaidis P Tziolas I Papoulidou F Prevalence, awareness, treatment and control of hypertension in employees of factories of Northern Greece: the Naoussa study J Hum Hypertens 2004 18 623 9 10.1038/sj.jhh.1001724 15029221

[B8] Psaltopoulou T Orfanos P Naska A Lenas D Trichopoulos D Trichopoulou A Prevalence, awareness, treatment and control of hypertension in a general population sample of 26,913 adults in the Greek EPIC study Int J Epidemiol 2004 33 1345 52 10.1093/ije/dyh249 15218014

[B9] Panagiotakos DB Pitsavos C Chrysohoou C Skoumas J Stefanadis C Status and management of blood lipids in Greek adults and their relation to socio-demographic, lifestyle and dietary factors: the ATTICA Study. Blood lipids distribution in Greece Atherosclerosis 2004 173 353 61 10.1016/j.atherosclerosis.2003.12.031 15064113

[B10] Gikas A Sotiropoulos A Panagiotakos D Peppas T Skliros E Pappas S Prevalence, and associated risk factors, of self-reported diabetes mellitus in a sample of adult urban population in Greece: MEDICAL Exit Poll Research in Salamis (MEDICAL EXPRESS 2002) BMC Public Health 2004 4 2 10.1186/1471-2458-4-2 15028111 PMC368436

[B11] Panagiotakos DB Pitsavos CH Chrysohoou C Skoumas J Papadimitriou L Stefanadis C Toutouzas PK Status and management of hypertension in Greece: role of the adoption of a Mediterranean diet: the Attica study J Hypertens 2003 21 1483 9 10.1097/00004872-200308000-00011 12872041

[B12] Gikas A Sotiropoulos A Panagiotakos D Pappas S Prevalence of self-reported myocardial infarction in a Greek sample: findings from a population-based study in an urban area (medical express 2002) Cent Eur J Public Health 2004 12 207 10 15666459

[B13] Vemmos KN Bots ML Tsibouris PK Zis VP Grobbee DE Stranjalis GS Stamatelopoulos S Stroke incidence and case fatality in southern Greece: the Arcadia stroke registry Stroke 1999 30 363 70 9933272 10.1161/01.str.30.2.363

[B14] Gikas A Sotiropoulos A Panagiotakos D Pastromas V Papazafiropoulou A Pappas S Prevalence trends for myocardial infarction and conventional risk factors among Greek adults (2002-06) Qjm 2008 101 705 12 10.1093/qjmed/hcn076 18603596

[B15] Athyros VG Mikhailidis DP Papageorgiou AA Didangelos TP Ganotakis ES Symeonidis AN Daskalopoulou SS Kakafika AI Elisaf M Prevalence of atherosclerotic vascular disease among subjects with the metabolic syndrome with or without diabetes mellitus: the METS-GREECE Multicentre Study Curr Med Res Opin 2004 20 1691 1701 10.1185/030079904X5599 15587481

[B16] Panagiotakos DB Pitsavos C Chrysohoou C Stefanadis C The epidemiology of Type 2 diabetes mellitus in Greek adults: the ATTICA study Diabet Med 2005 22 1581 8 10.1111/j.1464-5491.2005.01731.x 16241925

[B17] Pitsavos C Panagiotakos DB Chrysohoou C Stefanadis C Epidemiology of cardiovascular risk factors in Greece: aims, design and baseline characteristics of the ATTICA study BMC Public Health 2003 3 32 10.1186/1471-2458-3-32 14567760 PMC270056

[B18] Stergiou GS Thomopoulou GC Skeva II Mountokalakis TD Prevalence, awareness, treatment, and control of hypertension in Greece: the Didima study Am J Hypertens 1999 12 959 65 10.1016/S0895-7061(99)00136-3 10560781

[B19] Widimsky P Wijns W Fajadet J de Belder M Knot J Aaberge L Andrikopoulos G Baz JA Betriu A Claeys M Danchin N Djambazov S Erne P Hartikainen J Huber K Kala P Klinceva M Kristensen SD Ludman P Ferre JM Merkely B Milicic D Morais J Noc M Opolski G Ostojic M Radovanovic D De Servi S Stenestrand U Studencan M Tubaro M Vasiljevic Z Weidinger F Witkowski A Zeymer U Reperfusion therapy for ST elevation acute myocardial infarction in Europe: description of the current situation in 30 countries Eur Heart J 2010 31 943 957 10.1093/eurheartj/ehp492 19933242 PMC2854523

[B20] Weeks MF Kulka RA Lessler JT Whitmore RW Personal versus telephone surveys for collecting household health data at the local level Am J Public Health 1983 73 1389 94 10.2105/AJPH.73.12.1389 6638234 PMC1651264

[B21] Holbrook AL Green MC Krosnick JA Telephone Versus Face-To-Face Interviewing Of National Probability Samples With Long Questionnaires: Comparisons Of Respondent Satisficing And Social Desirability Response Bias Public Opinion Quarterly 2003 67 79 125 10.1086/346010

[B22] Hox JJ de leeuw ED A comparison of nonresponse in mail, telephone, and face-to-face surveys:Applying multilevel modeling to meta-analysis Qual Quant 1994 28 329 344 10.1007/BF01097014

[B23] Athyros VG Elisaf M Mikhailidis DP Undertreatment of dyslipidaemia in Greece Atherosclerosis 2004 177 215 6 10.1016/j.atherosclerosis.2004.06.021 15488888

