# Supplementary material for: Self-reported prevalence of atherothrombosis in a general population sample of adults in Greece; A telephone survey
Source: BMC Cardiovasc Disord. 2011 Apr 14;11:16. doi: 10.1186/1471-2261-11-16 (PMC3104943; doi:10.1186/1471-2261-11-16)
Supplement: Additional file 1 — Questionnaire. The file contains the questionnaire used to collect data. [file 1471-2261-11-16-S1.DOC]

**STUDY**

**Atherothrombosis in greece:**

**estimation of prevalence**

**athens march 09**

| **AREA OF RESIDENCE** | □ City………….………………………………..  □ Address………..………………………………….. |
| --- | --- |
|  |  |
| **GENDER** | □ Male  □ Female |
|  |  |
| **BIRTH DATE (Year)** |  |
|  |  |
| **NATIONALITY** | …………………………………….………….. |
|  |  |
| **OCCUPATION** | □ Unemployed  □ Retired  □ University student  □ Housewife  □ Servant  □ Free launcher  □ Farmer  _________________________________________________  □ Full time job  □ Part time job  □ Incapable of working |
|  |  |
| **MARITAL STATUS** | □ Married  □ Living together  □ Single  □ Divorced  □ Other _________________________________________ |
|  |  |
| **EDUCATIONAL STATUS** | □ Illiterate  □ Elementary school  □ Gymnasium  □ Lyceum  □ University  □ MSc  □ PhD  □ Other _________________________________________ |

| **OTHER** | □ Weight (kg)  ///////////////////////////  □ Height (cm)  □ Smoking      □ Number of cigarettes |
| --- | --- |

**Declare whether you have been diagnosed with any disease from the followings:**

|  |  | | |
| --- | --- | --- | --- |
| **Disease** | | **Date of diagnosis?**  **(Month – Year)** | **Treatment** |
| □ Diabetes Medicine  □ Hypertension  □ Hypercholesterolemia (i.e. high cholesterol level, drug-treated)  □ Claudication  □ Asymptomatic stenosis of peripheral vessel | | / | ______________________________________________________________________________________________________________________________________________________________________________ _________________________________________________________________________________________________________________________________________________ |

**Declare whether you have been diagnosed with any disease from the followings:**

| **Event** | **Date of diagnosis**  **(month – year)** | **Diagnostic tests?** |
| --- | --- | --- |
| □ Angina diagnosed by appropriate tests (i.e. angiocardiography, thallium, electrocardiogram) and which is treated with drugs  □ Myocardial infarction diagnosed by appropriate tests (i.e. angiocardiography, thallium, electrocardiogram) and which hospitalization was required  □ Transient ischemic stroke diagnosed by neurologist and was full rehabilitated  □ Ischemic stroke diagnosed by MRI or CT and for which hospitalization was required  □ Peripheral arterial disease (that is disease of carotids, nether limbs, aorta, etc) diagnosed by appropriate tests (i.e. angiography, CT, etc |  | _______________________________________________________________________________________________________________________________________________________________________________________________________________________________________________________________________________________________________________________________________________________________________________________________________________________________________________________________________________________________________________________________________________________________________ |

**Declare whether and when you have been subjected to the following interventions**

| **Intervention** | **Date of intervention?** | **Hospital** |
| --- | --- | --- |
| □ PTCI  □ CABG  □ Angioplasty of peripheral vessels  □ Surgery of peripheral disease | / | _________________________________________________________________________________________________________________________________________________________________________________________________________________________________________________________________________________________________________________________________________________________________________________________ |
